# Supplementary material for: Comparison of EMT-Related and Multi-Drug Resistant Gene Expression, Extracellular Matrix Production, and Drug Sensitivity in NSCLC Spheroids Generated by Scaffold-Free and Scaffold-Based Methods
Source: Int J Mol Sci. 2022 Nov 1;23(21):13306. doi: 10.3390/ijms232113306 (PMC9657250; doi:10.3390/ijms232113306)
Supplement: Supplementary file 1 [file ijms-23-13306-s001.zip › ijms-1962809-supplementary.pdf]

Supplementary Materials

Comparison of EMT-related and multi-drug resistant gene expression, extracellular matrix production, and drug sensitivity in NSCLC spheroids generated by scaffold-free and scaffold-based methods

Xiaoli Qi[1,2], Alexandra V. Prokhorova[1], Alexander V. Mezentsev[1], Ningfei Shen[1,2], Alexander V. Trofimenko[1], Gleb I. Filkov[1,2], Rushan A. Sulimanov[2], Vladimir A. Makarov[2], and Mikhail O. Durymanov[1,2*]

[1] Moscow Institute of Physics and Technology (National Research University), Dolgoprudny, Russia

[2] Yaroslav-the-Wise Novgorod State University, Veliky Novgorod, Russia

Supplementary Table S1. **Gene-specific primers used in the qPCR experiments**

Gene	Sequence ID	Name of the primer	Sequence of the primer	Product size, bp
IFNG	NM_000619.3	IFNG forward	GGTCATTCAGATGTAGCGGA	281
		IFNG reverse	TGCATCCTTTTTCGCCTTGC	
TNFA	NM_000594.4	TNF forward	GTTGTAGCAAACCCTCAAGC	148
		TNF reverse	TCACCCCGAAGTTCAGTAGA	
IL6	NM_000600.5	IL6 forward	ATGTGTGAAAGCAGCAAAGAGG	179
		IL6 reverse	TGCAAGTGCATCATCGTTGT	
IL1B	NM_000576.3	IL1B forward	GATGCACCTGTACGATCACT	229
		IL1B reverse	CACGGGAAAGACACAGGTAG	
IL12A	NM_000882.4	IL12A forward	CTCCTGGACCACCTCAGTTTG	89
		IL12A reverse	AGCTCCCTCTTGTTGTGGAA	
IL23A	NM_016584.3	IL23A forward	CTTCTCTGCTCCCTGATAGC	90
		IL23A reverse	TTCGAAGGATCTTGGAACGG	
IL10	NM_000572.3	IL10 forward	GCAAAACCAAACCACAAGACAG	194
		IL10 reverse	AGTCCTGCATTAAGGAGTCG	

TGFB	NM_000660.7	TGFB forward	TGGTGGAAACCCACAACGAA	113
		TGFB reverse	GTAGCGAAGTGCAGGTCAAT	
EGF	NM_001963.6	EGF forward	GGATTGACACAGAAGGAACCA	156
		EGF reverse	TCTATCCACTTCAGGGCTGT	
VEGFA	NM_001025366.3	VEGFA forward	CAGCTACTGCCATCCAATCG	165
		VEGFA reverse	AACACAAGTCCACAGCAGTC	
BFGF/FGF2	NM_002006.6	BFGF forward	ATGTAGAAGATGTGACGCCG	141
		BFGF reverse	AGTTCGTTTCAGTGCCACAT	
18SRNA	NR_003286.4	18S RNA forward	CAGCCACCCGAGATTGAGCA	253
		18S RNA reverse	GGACAGGACTAGGCGGAACA	
